# Disenrollment From Special Needs and Other Medicare Advantage Plans Among Nursing Home Residents

**DOI:** 10.1001/jamanetworkopen.2025.23973

**Published:** 2025-07-31

**Authors:** Hyunkyung Yun, Momotazur Rahman, Brian McGarry, Elizabeth M. White, David J. Meyers, Cyrus M. Kosar

**Affiliations:** 1 Department of Health Services, Policy & Practice, School of Public Health, Brown University, Providence, Rhode Island; 2 Division of Geriatrics and Aging, Department of Medicine, University of Rochester School of Medicine and Dentistry, Rochester, New York

## Abstract

This cohort study analyzes variations in Medicare plan disenrollment among long-stay nursing home residents from 2010 to 2022.

## Introduction

US nursing homes provide long-term care to approximately 1 million individuals with chronic illness and functional impairment. Although most residents have historically been enrolled in traditional Medicare (TM), enrollment in Medicare Advantage (MA) plans and MA special needs plans (SNPs) has grown considerably.^1^ However, evidence on the success of MA in long-term care settings is limited. We analyzed variation in plan disenrollment, a marker of enrollee experience and plan satisfaction,^2^ among nursing home residents enrolled in different Medicare plan types.

## Methods

This cohort study followed the STROBE reporting guideline and was approved by the Brown University institutional review board with a waiver of informed consent due to the use of deidentified data. Study data sources include Medicare enrollment files, the Minimum Data Set, and Care Compare (formerly nursing home compare). We identified Medicare beneficiaries residing in nursing homes for at least 100 days (ie, long-stays) between January 2010 and January 2022. We classified beneficiary coverage type in each year as TM, institutional SNP (I-SNP), dual-eligible SNP (D-SNP), or conventional MA plan. Chronic condition SNP enrollees were excluded due to low enrollment (<1%). We assessed 2 disenrollment measures: switches between major payers (ie, TM enrollees switching to any MA plan and vice versa), and switches between plan types (ie, D-SNP enrollees switching to other MA plans or TM). Enrollees were considered disenrolled if they had a different payer or plan type in January of the following year. When individuals died, disenrollment was based on coverage at the month of death. We estimated adjusted disenrollment rates using year-specific linear probability models with resident- and facility-level controls and state fixed effects. Sensitivity analyses were restricted to Medicare-Medicaid dual enrollees; however results were broadly consistent. We also characterized long-stay residents who switched to a different payer using 2019-2020 data. Analyses were performed using Stata version 18.0 (StataCorp) from December 2024 to May 2025. Significance was a 2-sided *P* < .05.

## Results

We identified 5 937 942 unique long-stay residents from January 2010 to January 2022 (3 803 976 female [64.1%]; mean [SD] age, 80.3 [11.9] years). Disenrollment rates varied across Medicare plan types (Figure). The risk-adjusted disenrollment rate for TM enrollees grew from approximately 1.7% (95% CI, 1.7%-1.7%) in 2010-2011 to 5.9% (95% CI, 5.8%-5.9%) in 2021-2022 (Figure A). Among conventional MA plan enrollees, disenrollment to TM fell from 22.0% (95% CI, 21.8%-22.3%) in 2010-2011 to 11.6% (95% CI, 11.5%-11.8%) by 2021-2022. Among I-SNP enrollees, annual disenrollment to TM ranged between 3.4% (95% CI, 3.2%-3.7%) to 5.2% (95% CI, 4.9%-5.4%). Among D-SNP enrollees, rates of disenrollment to TM ranged between 11.5% (95% CI, 11.0%-12.0%) to 16.0% (95% CI, 15.6%-16.4%). When intra–MA plan switching was accounted for (Figure B), disenrollment rates were approximately 4 to 5 percentage points higher for conventional MA and D-SNP enrollees and approximately 1 percentage point higher for I-SNP enrollees. Residents who switched payers during 2019-2020 were on average younger (mean [SD] age, 76.9 [11.9] vs 79.8 [12.0] years), less likely to be White (62 400 of 93 316 individuals [66.9%] vs 944 448 of 1 222 402 individuals [77.3%]), and less likely to have dementia (41 810 individuals [44.8%] vs 623 589 individuals [51.0%]) compared with residents who did not switch (Table). Residents who switched payers lived in nursing homes that were larger (mean [SD] number of beds, 153.1 [91.7] vs 137.1 [85.0]) and were more likely to receive care at a facility that was for-profit (72 420 individuals [78.4%] vs 850 643 individuals [70.2%]) and lower-rated (33 185 individuals [35.8%] vs 501 216 individuals [41.2%] at a facility with 4-5 stars).

**Figure.**
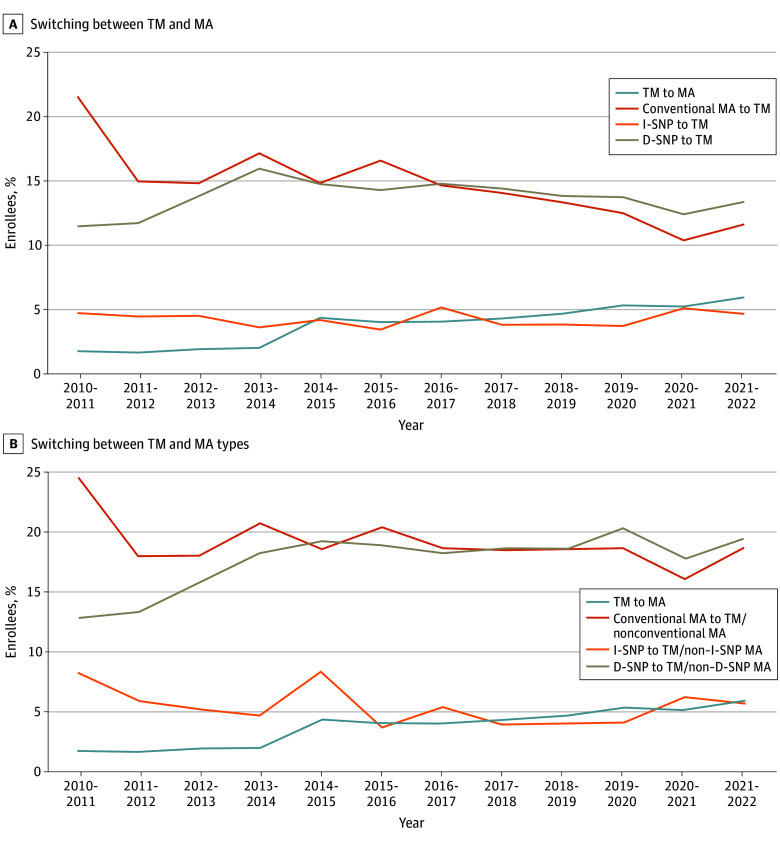
Risk-Adjusted Annual Medicare Plan Disenrollment Rates Among Long-Stay Nursing Home Residents, 2010-2021 In panel A, disenrollment was defined as either a traditional Medicare (TM) enrollee switching to Medicare Advantage (MA) or an MA enrollee of any plan type switching to TM in January of the following year. In panel B, disenrollment was defined as either a TM enrollee switching to MA or an MA enrollee of any plan type switching to TM or different type of MA in January of the following year. Risk-adjusted annual disenrollment rates were calculated using year-specific linear probability models that controlled for residents’ clinical and demographic characteristics, facility characteristics, and state fixed effects. D-SNP indicates dual eligible special needs plan; I-SNP, institutional special needs plan.

**Table.** Characteristics of Nursing Home Residents (2019-2020) Who Disenrolled From or Remained in Their Medicare Plan^a^

Characteristic	Participants, No. (%) (1 315 718)									
	Overall		Traditional Medicare		Conventional Medicare Advantage		I-SNP		D-SNP	
	Disenrolled (n = 93 316)	Remained (n = 1 222 402)	Disenrolled (n = 50 222)	Remained (n = 929 901)	Disenrolled (n = 31 004)	Remained (n = 207 776)	Disenrolled (n = 4163)	Remained (n = 52 267)	Disenrolled (n = 7927)	Remained (n = 32 458)
Age, mean (SD), y	76.9 (11.9)	79.8 (12.0)	76.3 (12.5)	79.5 (12.3)	78.5 (10.8)	82.0 (10.2)	75.3 (12.4)	78.9 (11.9)	75.0 (11.4)	77.2 (12.0)
Sex										
Female	57 602 (61.7)	789 540 (64.6)	31 145 (62.0)	594 910 (64.0)	19 194 (61.9)	136 753 (65.8)	2516 (60.4)	35 925 (68.7)	4747 (59.9)	21 952 (67.6)
Male	35 714 (38.3)	432 862 (35.4)	19 077 (38.0)	334 991 (36.0)	11 810 (38.1)	71 023 (34.2)	1647 (39.6)	16 342 (31.3)	3180 (40.1)	10 506 (32.4)
Race and ethnicity										
Asian or Pacific Islander	1828 (2.0)	25 634 (2.1)	810 (1.6)	19 862 (2.1)	598 (1.9)	3414 (1.6)	66 (1.6)	965 (1.8)	354 (4.5)	1393 (4.3)
Black	19 256 (20.6)	163 045 (13.3)	10 212 (20.3)	123 722 (13.3)	5558 (17.9)	22 450 (10.8)	1213 (29.1)	11 173 (21.4)	2273 (28.7)	5700 (17.6)
Hispanic	8608 (9.2)	71 171 (5.8)	3847 (7.7)	53 783 (5.8)	2952 (9.5)	10 359 (5.0)	370 (8.9)	3601 (6.9)	1439 (18.2)	3428 (10.6)
White	62 400 (66.9)	944 448 (77.3)	34 676 (69.0)	717 678 (77.2)	21 523 (69.4)	169 440 (81.5)	2454 (58.9)	35 928 (68.7)	3747 (47.3)	21 402 (65.9)
Other^b^	1224 (1.3)	18 104 (1.5)	677 (1.3)	14 856 (1.6)	373 (1.2)	2113 (1.0)	60 (1.4)	600 (1.1)	114 (1.4)	535 (1.6)
Medicaid enrollment	69 783 (74.8)	857 606 (70.2)	39 406 (78.5)	654 865 (70.4)	18 698 (60.3)	119 576 (57.6)	4044 (97.1)	50 888 (97.4)	7635 (96.3)	32 277 (99.4)
Heart failure	18 661 (20.0)	263 622 (21.6)	10 095 (20.1)	199 245 (21.4)	6068 (19.6)	46 015 (22.1)	906 (21.8)	11 690 (22.4)	1592 (20.1)	6672 (20.6)
Chronic lung disease^c^	22 103 (23.7)	279 491 (22.9)	12 089 (24.1)	213 910 (23.0)	6783 (21.9)	44 202 (21.3)	1169 (28.1)	13 132 (25.1)	2062 (26.0)	8247 (25.4)
Dementia	41 810 (44.8)	623 589 (51.0)	22 996 (45.8)	468 996 (50.4)	13 815 (44.6)	109 559 (52.7)	2118 (50.9)	31 186 (59.7)	2881 (36.3)	13 848 (42.7)
ADL score, mean (SD)	16.4 (6.0)	16.9 (6.2)	16.1 (6.1)	16.9 (6.3)	16.8 (5.7)	17.4 (5.5)	15.9 (7.0)	16.5 (6.6)	16.8 (6.0)	16.0 (6.4)
Facility attributes										
Total beds, mean (SD)	153.1 (91.7)	137.1 (85.0)	155.3 (92.3)	133.9 (82.6)	144.6 (86.1)	136.0 (77.8)	178.7 (100.5)	194.5 (122.8)	158.8 (100.3)	140.4 (89.2)
For-profit facility	72 420 (78.4)	850 643 (70.2)	38 385 (77.2)	652 986 (70.9)	24 325 (79.3)	138 428 (67.0)	3322 (80.7)	37 704 (72.6)	6388 (81.5)	21 525 (66.8)
Star-rating: 4-5	33 185 (35.8)	501 216 (41.2)	18 048 (36.2)	378 614 (41.0)	10 941 (35.6)	87 916 (42.5)	1420 (34.1)	22 077 (42.2)	2776 (35.3)	12 609 (39.0)

Abbreviations: ADL, activities of daily living; D-SNP, dual eligible special needs plan; I-SNP, institutional special needs plan.

^a^ Resident characteristics were obtained from the Medicare Beneficiary Summary File and Minimum Data Set. Facility-level characteristics were obtained form care compare report cards.

^b^ Defined as American Indian or Alaska Native, unknown, or any race or ethnicity not otherwise specified.

^c^ Indicated if a resident had either asthma or chronic obstructive pulmonary disease, a combined diagnostic checkbox of the Minimum Data Set.

## Discussion

This cohort study found that between 2010 and 2022, annual disenrollment rates from non-SNP MA plans fell, but remained high, particularly if intra—MA plan switching was accounted for. Disenrollment rates from TM increased but remained low. Disenrollment rates for D-SNPs and I-SNPs showed no obvious temporal trend. D-SNP disenrollment rates were high in this study, which may relate to their benefits being arguably more tailored to community-dwellers.^3^ By contrast, I-SNP disenrollment rates were low, which may indicate a higher degree of plan satisfaction.^2,4^ Those who disenrolled from I-SNPs and other plans did not have obviously different clinical characteristics. These results, combined with lower hospitalization rates previously documented, may reflect high-quality care for I-SNP enrollees.^5,6^ A limitation is that our analysis is cross-sectional and lacks data on reasons for plan switching; therefore, we cannot confirm the mechanisms underlying our findings. Nonetheless, our findings of high disenrollment in MA plans outside of I-SNPs warrant policy attention because they may signal unmet needs.

## Notes

Supplement.
Data Sharing Statement
