# Supplementary material for: Disenrollment From Special Needs and Other Medicare Advantage Plans Among Nursing Home Residents
Source: JAMA Netw Open. 2025 Jul 31;8(7):e2523973. doi: 10.1001/jamanetworkopen.2025.23973 (PMC12314716; doi:10.1001/jamanetworkopen.2025.23973)
Supplement: Supplement. — Data Sharing Statement [file jamanetwopen-e2523973-s001.pdf]

Data Sharing Statement

Yun. Disenrollment From Medicare Advantage Plans Among Long-Stay Nursing Home Residents. *JAMA Netw Open*. Published July 31, 2025.
doi:10.1001/jamanetworkopen.2025.23973

Data
Data available: No
